# Malnutrition and Dietary Habits Alter the Immune System Which May Consequently Influence SARS-CoV-2 Virulence: A Review

**DOI:** 10.3390/ijms23052654

**Published:** 2022-02-28

**Authors:** Ashmika Foolchand, Terisha Ghazi, Anil A. Chuturgoon

**Affiliations:** Discipline of Medical Biochemistry, School of Laboratory Medicine and Medical Science, College of Health Sciences, Howard College Campus, University of Kwa-Zulu Natal, Durban 4041, South Africa; ashmikafoolchand@yahoo.com (A.F.); terishaghazi@gmail.com (T.G.)

**Keywords:** gut microbiota, SARS-CoV-2, immunotoxicity, nutrition, diet, malnutrition

## Abstract

COVID-19, resulting from the SARS-CoV-2 virus, is a major pandemic that the world is fighting. SARS-CoV-2 primarily causes lung infection by attaching to the ACE2 receptor on the alveolar epithelial cells. However, the ACE2 receptor is also present in intestinal epithelial cells, suggesting a link between nutrition, virulence and clinical outcomes of COVID-19. Respiratory viral infections perturb the gut microbiota. The gut microbiota is shaped by our diet; therefore, a healthy gut is important for optimal metabolism, immunology and protection of the host. Malnutrition causes diverse changes in the immune system by repressing immune responses and enhancing viral vulnerability. Thus, improving gut health with a high-quality, nutrient-filled diet will improve immunity against infections and diseases. This review emphasizes the significance of dietary choices and its subsequent effects on the immune system, which may potentially impact SARS-CoV-2 vulnerability.

## 1. Introduction

Since its outbreak in China, the coronavirus disease 2019 (COVID-19), caused by severe acute respiratory syndrome coronavirus 2 (SARS-CoV-2), has dramatically disseminated throughout the world. This highly pathogenic positive-sense RNA virus consists of structural proteins, namely spike, membrane, nucleocapsid and envelope proteins [1,2]. A vital factor of viral pathogenesis involves the angiotensin-converting enzyme-2 (ACE2) cellular receptor [3], which facilitates entry of the virus into susceptible cells. The receptor-binding domain of the spike protein binds to the ACE2 receptor activating membrane fusion of the virus to the host cell. Subsequently, viral RNA is released into the cytoplasm, and the infection is established [4]. Interestingly, apart from the lungs, the ACE2 receptor is also expressed in the kidney, gastrointestinal tract [5] and the enterocytes of the ileum and colon [6]. COVID-19 mainly targets the respiratory tract; however, it may fatally progress to multiple organ failure [7]. Although effective vaccines are now available, they do not provide 100% protection against COVID-19 infection; therefore, other intervention strategies should be explored to reduce the severity of the disease. Since certain foods demonstrate beneficial immune responses to respiratory viruses, the diet may be proposed to ease the adverse health consequences of COVID-19 [8,9].

The consequence of viral infections is highly dependent on the host’s nutritional status, as the body is exposed to a significant energetic effort to endure the defences [10]. The gut and commensal microbiota can regulate and be regulated by invasive infections, inducing a positive or suppressive result on the host [11]. To overcome the response to pathogens, a healthy gut microbiome is vital for maintaining an optimal immune system and avoiding immune responses that can prove deleterious to the lungs and other organs [12]. Therefore, it may be plausible to consider the gut for a solution to mitigate SARS-CoV-2 infection. It is also evident that the intestinal tract is a COVID-19 infection target as some infected patients present with vomiting and diarrhoea [13], while infected patients recently presented the SARS-CoV-2 RNA in their stool samples [14].

Diet maintains a vital role in human health as it can either affect the gut microbiota by altering physiological responses of the host or by directly attacking the host response [15]. The complex and dynamic mammalian gut microbial community is important for the maturation and development of mucosal and systemic immune responses [15]. The interplay among the microbiota, consumed nutrients and the immune system thus serve as regulators for homeostasis maintenance and protection from invasive pathogens [15]. During the infection process, it is presumed that the enterocytes are infected, thereby compromising the function of intestinal membranes. The intestine acts as a barrier to inhibit microorganisms and its products from leaking into the bloodstream, which is associated with the cytokine storm [16]. Bacteria in the gut produce pathogen-activated molecular patterns (PAMPs), which induce different immune responses via Toll-like receptors (TLRs) [17], depending on the cell, ligand or receptor type [12]. Inflammatory cytokines are released during SARS-CoV-2 injury, leading to a cytokine storm, which initiates an immune dysregulation through T cells and inflammatory monocytes [18]. Cytokines regulate the body’s response to infections and inflammation, and the production of cytokines is impacted by the gut microbial metabolic processes [19]. Modern lifestyles, which include sleep deprivation, daily stress and unbalanced diets, can influence the onset of a chronic low-inflammation, affecting the immune system negatively [20]. The highest COVID-19 morbidity and mortality are in the elderly, especially those with underlying health conditions related to inflammation and other disorders such as cardiovascular disease and diabetes [21]. Additionally, individuals with these underlying health conditions show a less diverse gut microbiome [22], suggesting a link between aging and shifts in gut diversity and pro-inflammatory states. Nutrition is also directly linked to inflammation and subsequently to immune responses. Malnutrition is a global problem that should not be ignored during the COVID-19 pandemic [23,24]. In malnutrition, the consumption of monotonous diets, abundant in highly processed foods, renders inadequate vitamins and minerals to the host, weakening the immune system and enhancing SARS-CoV-2 susceptibility [10]. Hence, controlling the inflammatory response may, therefore, be a potential strategy to combat the virus.

This review outlines existing literature on the effects of various diets and nutrient consumption on the immune responses to viral pathogens such as the SARS-CoV-2 infection.

## 2. Regulation of the Gut Microbiota

**Figure 1:**
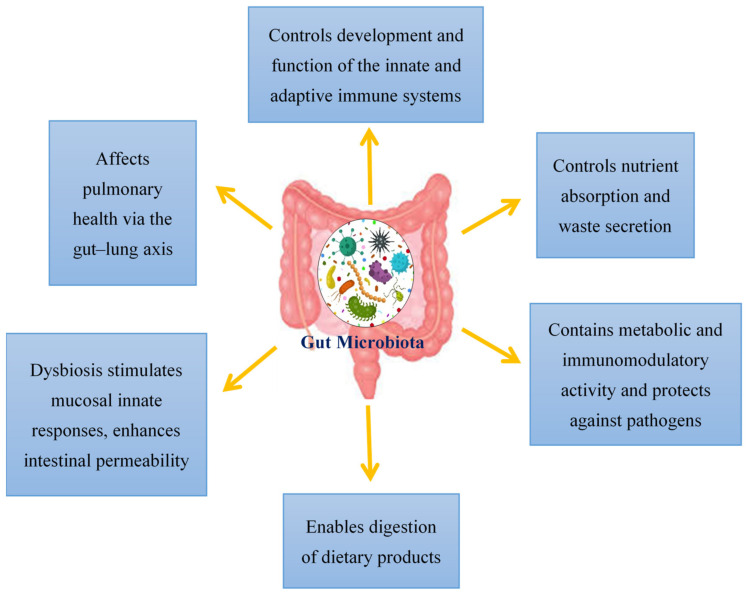
An overview of the functions of the gut microbiota in the host.

Viral infections have mostly been documented in terms of the virus, the host cell and the host immune system. However, over the past decade, viral infections have been affiliated with the term “microbiota revolution”, which links several pathological manifestations to the gut microbiota and its alterations [25]. The microbiota is a complex group of microorganisms that colonize the mucosal surfaces and are responsible for nutrient absorption and waste secretion [25]. The human gut microbiota contains 1014 resident microorganisms, including fungi, viruses, bacteria and archaea [26]. These microorganisms perform a vital role in health and disease attributing to its metabolic and immunomodulatory activity and protection against pathogens [27]. Commensal bacteria are particularly important for shaping the host immune system and triggering its responses [28,29]. The gut controls the formation and action of the adaptive and innate immune system by tuning immune cells for inflammatory responses and maintaining immune homeostasis [30]. This affects the host’s susceptibility to various diseases; therefore, in SARS-CoV-2 infection, a healthy gut microbiota is crucial for sustaining an optimum immune system which averts uncontrolled inflammatory responses [30]. Modifications of the gut microbiota are characterized by multiple factors, with the main cause being viral infections [25]. Additionally, the gut microbiota also affects pulmonary health via a crosstalk of the lungs and gut microbiota, known as the gut–lung axis [31]. This gut–lung axis is bidirectional; therefore, when microbial endotoxins affect the lungs via the blood causing inflammation, and the gut microbiota may also be affected [32]. The hosts gut microbiota enables the digestion of various dietary products. Dysbiosis of the gut microbiota stimulates mucosal innate immune responses and enhances the permeability of the intestine. This leads to the transfer of pathogenic organisms, allowing detrimental metabolites to access the intestinal epithelium and promoting disease severity [33]. Additionally, a link between ACE2 and the gut microbiota has also been documented. In a mouse model, ACE2 deficiency impaired tryptophan homeostasis, which altered the gut microbiome and inflammatory response [34]. In intestinal epithelial cells, the ACE2 receptor may also control nutrient uptake by attaching to amino acid transporters, suggesting that SARS-CoV-2 may compete against protein nutrients and disrupt absorption via the ACE2 receptor [35,36]. This brings about a possible link between SARS-CoV-2 infections and how the gut microbiota may impact infection severity (Figure 1).

## 3. The Role of ACE2 in the Gut

The type I membrane-anchored glycoprotein, ACE2, encloses 805 amino acids and contains an N-terminal peptidase domain along with a C-terminal collectrin-like domain [37]. ACE2 is a negative regulator of the Renin–Angiotensin System (RAS), thus providing relief from the harmful effects facilitated by angiotensin (Ang) II signalling via the Ang II receptor type I (AT1R) [38]. ACE2 also demonstrates RAS-independent roles which promote intestinal dysbiosis through loss of ACE2 expression or function [39]. This supports the gastrointestinal symptoms experienced by COVID-19 patients [40]. The gut microbiota also controls ACE2 expressions and therefore plays a role in COVID-19 severity and contagion [41].

**Figure 2:**
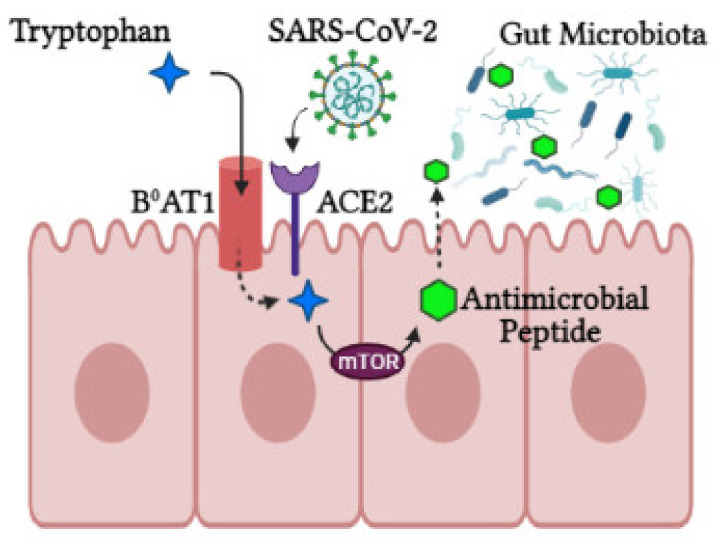
The role of ACE2 in the intestine. ACE2 is required for B0AT1 amino acid transporter that mediates tryptophan uptake. Tryptophan triggers antimicrobial peptide secretion via the mTOR pathway, which can alter the gut microbiota composition. Upon SARS-CoV-2 infection, ACE2 is downregulated, leading to aberrant absorption of tryptophan and antimicrobial peptides. This subsequently alters the gut microbiota, conferring susceptibility to inflammation (created with BioRender.com, accessed on 25 October 2021).

Activity of intracrine gut ACE2 encompasses modulation of electrolyte homeostasis, gastrointestinal epithelial fluid, gastrointestinal mucosal inflammation, smooth muscle control and gut-specific fibrosis [42]. In the gut, ACE2 shares a site with B^0^AT1 and functions as a membrane trafficking chaperone of B^0^AT1, which controls the sodium-dependent uptake of tryptophan and glutamine (neutral amino acids) into intestinal cells [41]. Animal ACE2-knockout studies displayed altered gut microbial composition, decreased tryptophan serum levels and reduced small intestinal antimicrobial peptide (AMPs) expression [34] in addition to reduced AMP results in dysbiosis, enhanced pathogen levels and impaired gut microbiota [43]. It is proposed that AMP expression and composition of the gut microbiota are regulated by mTOR activation via the tryptophan-nicotinamide pathway and/or nutrient sensing (Figure 2) [34,39].

During SARS-CoV-2 infection, luminal ACE2 levels are downregulated, impacting gut permeability, nutrient transport and local and systemic inflammation. In the luminal surface of enterocytes, ACE2 deficiency enhances Ang II and reduces Ang1-7. This Ang1-7 decrease in turn activates AT1R and increases gut permeability linked to leaky gut syndrome [39], which may facilitate a cytokine storm [44]. In addition, ACE2 deficiency downregulates ACE2-B^0^AT1 complexes, hindering neutral amino acid uptake, which are critical for T-cell function, Toll-like receptor signaling and NF-kβ activation and inflammation [44]. Tryptophan also triggers incretins, which regulate glucose homeostasis and promotes hypoglycemia. Furthermore, loss of ACE2-Mas receptor binding in the gut halts glucose transport mediated by SGLT1 and GLUT luminal glucose transporters [41]. In enterocytes’ luminal surfaces, ACE2 deficiency also involves digestive enzyme degradation to produce free amino acids [41].

## 4. The Influences of Various Diets and Nutrient Consumption on Viral Infections

Various dietary plans are known to effect the gut microbiota compositional patterns [12]. The gut contains an equilibrium of bacterial species, some of which are required for digestion of dietary fibres [45], while others produce essential nutrients [46]. The current understanding of how the microbiome is altered by dietary fat involves TLR4-mediated inflammation, resulting in impaired immune cell membrane function and shifts in nutrient availability [47,48]. Dietary simple sugars may cause dysbiosis via fluctuations in nutrient concentrations and bacterial functions—thus favouring injurious taxa [46,49,50]. Preliminary animal and culture-based research has demonstrated the ability of the gut microbiome to digest artificial sweeteners, which are deemed non-caloric for humans. Sweeteners can be metabolized into short-chain fatty acids (SCFA) by gut bacteria, which carry various consequences [51]. Although some SCFAs may be favourable, their production may alter the bacterial equilibrium [52,53], activate the TLR4 pathway and/or be converted to absorbable by-products that produce calories [51,54]. In vitro studies propose that processed, simple sugars also enhance blood inflammatory cytokine markers and decrease white blood cell phagocytosis [55]. A high fibre diet increased SCFA levels in the intestine and blood, and decreased lung injury from the respiratory syncytial virus infection [30]. The same effect was seen in mice fed water supplemented with acetic acid [56,57]. A westernized diet, classified by excessive ingestion of red meat, processed food and sugary beverages along with minimal fruit, vegetable and fibre intake, increases the frequency of metabolic diseases like diabetes and obesity, which are linked to systemic low-grade inflammation [58,59] (Table 1). Commonly consumed in developing countries, the western diet is also high in saturated fatty acids, which poses the risk of impairing the adaptive immune system while chronically activating the innate immune system [60]. Wild type mice given diets high in sucrose and fat presented reduced gut microbiota diversity and increased sensitivity to opportunistic pathogens, leading to a reduced occurrence of specific gut barrier protective bacteria [61]. Additionally, enhanced lung tissue macrophage infiltration, specifically in the alveoli, was observed in mice fed a diet high in saturated fat [62]. This is particularly applicable to COVID-19 patients, owing to the role of alveolar damage, inflammation of lung tissue and the increased infection rate in alveolar lung epithelial cells in COVID-19 pathology [63]. In humans, the comparison of the microbial shifts between a vegan, vegetarian and omnivore diet showed a significant increase in β-diversity within 24 h of changing to an animal-derived diet [64]. While saturated fats are highly inflammatory [65,66], excess omega-6 poly-unsaturated fats, found in cooking oils, are implicated in immune responses via various mechanisms including effects on TLR4 [67] and acting as precursors for inflammatory mediators [68,69]. Polyunsaturated fatty acids (PUFAs) are key inflammatory and adaptive immunity mediators, of which omega-3 and omega-6 stimulate anti- and pro-inflammatory effects [70]. Omega-3 fatty acids include eicosapentaenoic acid (EPA) and docosahexaenoic acid (DHA) that are known to possess beneficial immunity and inflammation effects. These PUFAs are enriched in fish oils and aid in the production of strong bioactive anti-inflammatory lipid mediators, namely protectins, resolvins and maresins [71]. In a study, the DHA-derived lipid mediator protectin D1 attenuated nucleoprotein mRNA expression of the influenza virus by more than 30%, thus impairing viral replication. Additionally, influenza infected mice were relieved from death upon co-treatment of protectin D1 with peramivir, 48 h after the infection [71]. In another study, DHA and EPA displayed potent anti-Hepatitis C virus activities at 100 µM [72]. Furthermore, based on an expert statement by the European Society for Parenteral and Enteral Nutrition, omega-3 fatty acids may be employed in COVID-19 patients to improve oxygenation [73]. On the contrary, others have expressed caution over the use of omega-3 in COVID-19 patients as evidence showed that increased susceptibility to cellular membrane damage promoted oxidative stress and inflammation [74]. Despite these findings, validated trial data are required for the use of omega-3. Several foods have antioxidant properties [75] and may interact with transcriptional factors that have antioxidant effects, such as nuclear factor erythroid 2–related factor 2 (Nrf2) [9]. Nrf2 can be activated by natural compounds in vegetables, fungi, plants and micronutrients [76], as well as foods containing *lactobacillus* [77]. Fermentation processes can promote the antioxidant activity of cereals, milk, fruit, fish, vegetables and meat [78] (Table 1). In rural households and village communities, fermented foods like alcoholic drinks, bread, vegetables and cheese have been made and consumed by millions of people for several years [79]. Fermented foods are produced by enzymatic conversions in food and controlled microbial growth [80]. Fermented foods contain live microorganisms and are known to regulate the intestinal microbiome [80,81,82]. Throughout the world, the composition of the microbiome varies among different regions. Genetic predisposition and diet contribute to the inter-individual variability of the gut microbiota, whereas *lactobacillus* spp. contributes to its diversity and regulates the gastrointestinal tract via oxidative stress [79]. In sub-Saharan Africa, commonly consumed fermented foods include maize, millet, sorghum, fruit and vegetables, some of which make up over 50% of the diet [79]. Kimchi, a staple Korean dish, is made from several fermented vegetables and shows anti-diabetic properties [83]. In vitro and in vivo studies have classified kimchi and sauerkraut as functional foods due to their anti-obesity, anti-constipation and anti-cancer characteristics alongside their ability to improve the immune system [84] (Table 1). Plant-based fermented beverages such as vinegar and kombucha, made from sugared tea, possess several health benefits. Vinegar has anti-obesity, anti-diabetic, antioxidant, anti-hypertensive and anti-microbial properties [85], while the ancient beverage kombucha shows anti-cancer, antioxidant and anti-microbial activity [86]. Additionally, the homemade fermented milk drink, kefir, regulates host immunity and decreases susceptibility to bacterial and viral infections [87]. The Mediterranean diet is known to reduce insulin resistance [88] (Table 1), in which Nrf2 seems to play a role [89]. The European Food Safety Authority performed an ecological study to determine the impact of fermented foodstuffs in COVID-19 mortality. These foods included fermented pickled vegetables, vegetables, milk, yoghurt and sour milk [90]. Among all the foods considered, only fermented vegetables showed a statistically significant COVID-19 mortality rate. Interestingly, for each gram per day increase in fermented vegetable consumed, the COVID-19 death risk was decreased by 35.4% [90]. In an ecological study, the outcomes of cruciferous vegetables, such as broccoli, cauliflower, leafy brassica and head cabbage (white, red and savoy cabbage) were compared to courgette, spinach, cucumber, lettuce and tomato [90]. Among these, head cabbage and cucumber were the only vegetables that showed statistical significance in COVID-19 mortality rate. For each gram per day increase in head cabbage and cucumber consumed, COVID-19 mortality risk decreased by 11–13.6% [90]. Western diets usually lack fermented foods and urbanization in western countries has resulted in changes to the gut microbiome and reduced intestinal diversity [91,92]. In Japan, westernized diets led to microbiome and insulin resistance changes [93]. Consumption of fast foods are characterized to reduce lactobacilli, which is essential for the breaking down of food, nutrient absorption and fighting diseases, in the microbiome [94]. Frequent snacking especially in between meals could lead to gut dysbiosis; therefore, it should be minimized or rather consist of fruit and vegetables [19].

Vitamins play a role in adaptive and innate immune reactions, with vitamin A and D being primary contributors [95]. Vitamin A sustains T-cell growth while vitamin D maintains antibody-secreting cell functions [96] (Table 1). In hypo-nutritional states, immune dysfunction is related to deficiencies of these vitamins, resulting from monotonous diets that are low in vitamin sources. Among these, other micronutrients also play a role in the immunocompetency of the host against infections, which include vitamins C, E, zinc, iron and selenium [97]. Vitamin A is abundantly found in carrots, sweet potatoes and green leafy vegetables [98]. Although there is minimal information on its role in preventing COVID-19, Briguglio, Pregliasco [10] reported that a vitamin A deficit is predominant in individuals with malnutrition, increasing their vulnerability to SARS-CoV-2 infection. Owing to its anti-inflammatory and antioxidant action, the maintenance of vitamin C levels in the body is vital for protection against pulmonary infections [99] (Table 1). Vitamin C can be acquired in the diet from oranges, strawberries, mango, red peppers, broccoli, lemon and vegetables [98]. Early intravenous and oral administration of high doses of vitamin C has been recommended for COVID-19 treatment and can be used as a preventative measure without any adverse side effects [100]. Vitamin D levels can be affected by limited exposure to the sun and outdoor physical activities [101]. Vitamin D has anti-inflammatory effects (Table 1); thus, administration of this vitamin lowers pro-inflammatory cytokine expressions and increases anti-inflammatory cytokine expressions [102] and may, therefore, effectively suppress the COVID-19 cytokine storm in patients [103]. Due to its protective properties against lung injury and acute respiratory infections, vitamin D2 or D3 supplementation may be used as an approach to inhibit moderate and severe respiratory infection symptoms like those seen in COVID-19 patients [104]. Vitamin E deficiency is observed in malnutrition and obesity and may be a factor for SARS-CoV-2 susceptibility [10]. Vitamin E is primarily acquired from vegetable oils, nuts, seeds, broccoli and spinach, and has been shown to have antioxidant and anti-inflammatory characteristics [105] (Table 1). Vitamin E also plays a role in respiratory tract infections [23]. A study displayed that men with higher serum α-tocopherol (a type of vitamin E) showed reduced mortality from respiratory diseases [106].

Meat, beans, eggs and dark green leafy vegetables provide a source of iron [99]. The link between iron and infections is not yet fully elucidated; however, some studies have documented that iron deficiency predisposes individuals to infections [107], while other studies suggest a protective role [108]. Therefore, the maintenance of iron homeostasis seems prudent in preventing COVID-19. Zinc is another important dietary compound required for optimal immune function, development and maintenance of immune cells [99]. Zinc controls pro-inflammatory responses via NF-κβ while its deficiency alters inflammatory responses (Table 1), increasing inflammation and damage to host tissue [109]. Individuals with insufficient zinc intake show a higher occurrence of obstructive lung disorder [99]. Recently, zinc was identified as a COVID-19 adjuvant, owing to its ability to regulate antiviral immunity and reduce inflammation [110]. For SARS-CoV-2 treatment, certain molecules were examined to prevent the viral enzyme RNA-dependent RNA polymerase affinity. These molecules serve as zinc ionophores, prevent mRNA capping, block elongation of RNA polymerase and promote mutations in viral replication [111,112], thereby acting as a SARS-CoV-2 antagonist. Zinc is sourced in foodstuffs of the Mediterranean diet, including beans, lentils, nuts, sesame, pumpkin seeds, red meat and poultry [99]. Among the Chinese population, preliminary reports indicate a positive link between selenium and COVID-19 cure rates [70], and this is consistent with previous studies showing the antiviral effects of selenium [113]. However, tests are still underway to find the suitable selenium dose required for COVID-19 prevention [99].

Probiotics are a promising tool in clinical research that have been proposed for the use in several pathological conditions. Probiotics contain live organisms; therefore, when administered in adequate amounts, it confers positive effects to patients [114]. Although not fully proven, probiotics has been recommended to combat viral infections and protect the host [115]. Attachment of the virus to the host cell is an important step in infections. Probiotic bacteria may potentially attach to the virus directly, thereby preventing the infectious process [25]. Reports show that lactobacilli can bind to and inactivate viruses via adsorptive and/or trapping mechanisms [25]. Lactobacilli contain immunomodulatory properties and protect from infections by promoting cytokine antiviral responses in the intestinal mucosa, immune cells and respiratory cells [116,117]. Administration of lactobacilli via the nose proved effective against viral respiratory infections, inducing innate immune responses in the epithelium of the airways [118]. In mice, *lactobacillus* improved defence against respiratory infections by inducing respiratory immune responses and increasing inflammatory signals [119]. Furthermore, *lactobacillus casei* promotes killing and phagocytosis in alveolar macrophages, which increases expressions of IgA, IFN-γ and TNF-α, thereby aiding the hosts battle against influenza virus [120]. Supplementation of probiotics has been proposed as a complementary remedy for gastrointestinal symptoms and to lower secondary COVID-19 infection risks from microbial translocation in acute cases [121]. Direct evidence of probiotics in COVID-19 treatment is yet to be proven; however, it is suggested that probiotics could serve as a complementary treatment to reduce SARS-CoV-2-induced inflammation and repair damaged intestinal mucosa by modulating the gut microbiota [25].

Another major concern is that the detrimental effects of diet can be passed on to future generations. Maternal diets may shape a child’s flavour preferences, skewing their palette to foods that could influence the tendency towards obesity and unhealthy diets [122]. Furthermore, children also inherit their microbiome from their mother. When the maternal diet has a harmful bacterial imbalance, this imbalance is passed to the child, failing to provide the ideal commensals for immune education during the child’s developmental period [47].

Although research is limited on the direct impact of food choices in COVID-19 prognosis, it is evident from the aforementioned data that diet plays a key role in host immunity. While westernized, sugar rich and high fibre diets impose a negative outcome on immune responses, diets rich in fruit, vegetables, fermented foodstuff, vitamins and probiotics are beneficial for efficient immune function against viral infections. Taking these findings into consideration, we propose that diet could be a key target in the combat and prevention against COVID-19 infections. However, extensive research is required to confirm the direct links between the diet and COVID-19.

## 5. The Effects of Dietary Choices on ACE2 Expression

A crucial step in SARS-CoV-2 viral entry involves the viral protein attaching to the host cell receptor. The human ACE2 receptor mediates SARS-CoV-2 viral entry as it binds with high affinity to the SARS-CoV-2 spike protein, resulting in the viral envelope and the host cell membrane merging together [123]. Emerging research has demonstrated that the *ACE2* gene function and expression can be impacted by dietary intake [124,125]. Preliminary studies have examined the effects of diets rich in fructose on ACE2 protein levels [126] and the effects of high dietary sodium on ACE2 receptor expression [127]; however, this has only been tested in infants. Other research determined the effect of dietary fat consumption on ACE2 expression. Among these, the dietary fat content in high-fat diets varies from 50–60% of total energy consumed [125,128,129]. In a 10-week mice study, *ACE2* gene activity was determined in a control group (10% lipids, 14%protein, 76% carbohydrate) versus a high-fat diet group (36% carbohydrates, 14% protein, 50% lipids). Upon assessing the mice liver, it was found that *ACE2* levels were decreased in the high-fat diet compared to the control [128]. Similarly, research in retroperitoneal adipose tissue of postnatal rats showed a decrease in *ACE2* gene expression after being fed a high-fat diet [125]. Additionally, a diet rich in fats in male mice led to decreased kidney ACE2 activity, while the ovariectomy of female mice fed a high-fat diet resulted in decreased adipose ACE2 activity [129]. There is also consistent research on the role of resveratrol to influence ACE2 expression. This polyphenolic compound is present in plant-derived foods such as grapes, berries, cocoa and red wine, and is known for its protective role in cardiovascular disease, cancer and respiratory illnesses [130,131]. Rats fed a resveratrol diet (50 mg·kg^−1^/day) displayed upregulated ACE2 protein expression [132]. In another experiment, mice given a combined diet of high-fat and resveratrol significantly upregulated *ACE2* expression, in contrast to mice fed a diet high in fat alone [124]. This suggests that dietary resveratrol may assist in preventing the harmful impacts of a high-fat diet on the *ACE2* mRNA expression [124]. In a human in vitro study, aortic smooth muscle cells incubated for 24 h with resveratrol displayed a significant increase in ACE2 gene and protein expressions [133].

## 6. The Risks Imposed by Malnutrition

**Figure 3:**
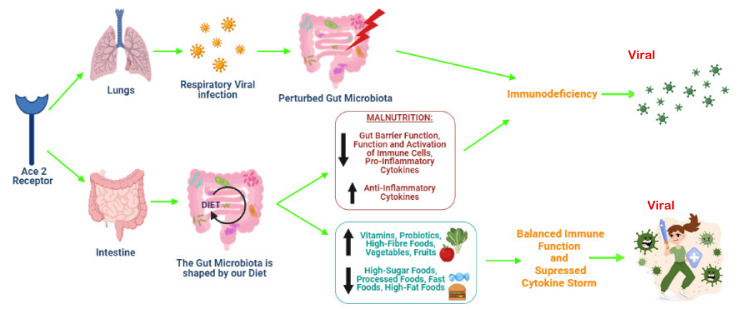
The impact of various nutrients and foodstuffs on the immune system of the host (created with BioRender.com, accessed on 25 October 2021).

Worldwide, malnutrition is the main cause of immunodeficiency [134] as it alters both the adaptive and innate immune responses which protect against viral proliferation [135]. Chronic diseases, considered as contributors of severe COVID-19, are frequently linked to protein–energy malnourishment, impairing immune cell activation [136], thereby permitting viral persistence and enhanced inflammatory cell transfer to the lungs [137]. Increased metabolism and extreme nitrogen loss are allied with infectious states; therefore, malnourished persons are disadvantaged due to reduced body reserves [10]. For instance, mice fed protein, zinc and iron in amounts lower than the optimal requirement encountered a drop in effector CD4^+^ T cells and body weight as compared to normal nourished mice [138]. Various researchers have documented that malnutrition alters immune responses [139], with the most evident change being the functional and structural involution of the thymus, thereby decreasing the T cell response [139,140]. Research also reveals that malnutrition hinders phagocytic functions and altered cytokine and antibody production [141]. Despite the robust relation between infections and malnutrition, the mechanisms driving this association are not entirely understood [142,143]. This limited understanding may be due to the complex interactions between nutrition and infections, which lead to a vicious cycle [144]. During this vicious cycle, infections prompt an inflammatory response, resulting in fever, appetite loss, increased catabolism and intestinal absorption anomalies. These modifications enhance nutritional needs and initiate or aggravate malnutrition [143]. Malnutrition then reduces the gut barrier function and increases the chance of infections [143,145]. This modifies the intestinal microbiota [146] and compromises the activation and generation of immune cells, altering inflammatory adipocytokine regulation and limiting macro- and micronutrient uptake [147,148]. Undernutrition decreases lipid tissue, impairing adipokine production and inducing innate and adaptive immunity restrictions. In malnourished states, production of leptin was downregulated while adiponectin production was upregulated [99]. Adiponectin enhances alternative macrophage activity and secretion of anti-inflammatory cytokines, lowering T cell responses and production of B cells [99]. In addition, the pro-inflammatory responses of immune cells are limited by impaired production of stress hormones in combination with downregulated leptin and upregulated adiponectin production in malnutrition [148]. Ultimately, in malnutrition, pro-inflammatory cytokines (TNF-α, IL-6, IL-8), which are essential for killing pathogens, are reduced, while anti-inflammatory cytokines (IL-10, IL-33) are increased [148]. The prevalence of severe diseases was increased in undernourished children with lower respiratory tract and respiratory syncytial viral infections [149]. Experimental studies on influenza infections revealed that energy and protein malnutrition also prompted the risks of acute infections [137], decreasing virus-specific antibodies and responses of CD8^+^ T cells [137]. In a cross-sectional study with COVID-19 patients, Li, Zhang [150] indicated that the elderly are often malnourished. They revealed that 27.5% of patients, aged over 65 years, were in danger of malnutrition, while 52.7% were already malnourished [150]. In these cases, the fear of contracting COVID-19, prolonged social isolation during lockdown and the yearning to resume normal routine life caused anxiety, which compromises appetite and promotes malnutrition [150]. Currently, little is known about the impact of malnutrition in COVID-19 patients; however, symptoms such as breathlessness, loss of taste and smell, hyper-metabolism, vomiting and diarrhea present in infected patients may result in malnutrition and loss of body weight [151]. One of the prime roots of immunodeficiency is malnutrition, with 38–78% of ICU patients being malnourished [152]. A study assessing malnutrition in 114 COVID-19 patients revealed that 47 patients were malnourished, with ICU patients showing a significantly high prevalence of malnutrition (66.7%) [151]. In a similar study, Rouget, Vardon-Bounes [153] found that 30 out of 80 admitted COVID-19 patients were malnourished, of which 70% presented severe malnutrition. Furthermore, Abate, Chekole [154] summarized the findings from 14 studies reporting the prevalence of malnutrition in hospitalized COVID-19 patients. Among 4187 hospitalized COVID-19 participants, meta-analysis revealed a pooled prevalence of 49.11% malnutrition, with the highest prevalence seen in the critically ill patients [154]. A retrospective study in China consisting of 139 patients revealed that malnutrition also contributed to prolonged hospitalization of COVID-19 patients [155]. In a cohort of 136 severely ill, ICU admitted COVID-19 patients, 61% had a high nutritional risk [152]. Similarly, 77% of an Italian cohort of elderly hospitalized COVID-19 patients were at nutritional risk, while 50% were malnourished [156]. In France, 42.1% of non-ICU patients were identified with malnutrition, whereas the prevalence of malnutrition in ICU admitted patients reached 66.7% [151]. In Morocco, 14.6% and 65.9% of ICU admitted COVID-19 patients presented malnutrition and were at nutritional risk, respectively [157] (Figure 3).

## 7. Conclusions

The COVID-19 pandemic imposes a social and economic impact on the world. Social isolation, although being an effective strategy to avoid the spread of the virus, causes anxiety and depression from limited interaction with friends and family. In such situations, sleep deprivation, everyday stress and unbalanced diets induce chronic inflammation that adversely impacts the immune system. Although there are safe and efficient vaccines, it is necessary to explore other potentially useful approaches to ameliorate disease severity. Generally, inflammatory stimuli include viral and bacterial infections, endocrine, toxic, genetic and metabolic factors. However, diet and lifestyle may influence inflammation and subsequently alter functions of the immune system. Research on the gut microbiota has expanded our understanding on infectious and chronic diseases. The diversity of the gut microbiota and its residing beneficial microorganisms may determine the course of infections and diseases. A balanced nutritional status and healthy eating choices are important to manage viral infections, such as those triggered by SARS-CoV-2 in malnutrition. Although there are limited data on the nutritional management of SARS-CoV-2 infection, interventions must be made to decrease inflammation and strengthen the immune system. Some recommendations to enhance host nutrition include increased vitamins, probiotics, high-fibre food, vegetable and fruit consumption but minimized consumption of high-sugar food, processed food, fast foods and high-fat foods, to balance the immune function and suppress the cytokine storm. In addition to the conventional COVID-19 control measures such as social distancing, wearing a mask and sanitizing, it is proposed that fermented vegetables and foods with antioxidant properties may help limit infection severity. While nutrition is a promising tool for COVID-19 management, a better understanding concerning nutrition and SARS-CoV-2 is imperative, as the pathogen fitness might depend on host resource availability. Hence, the relationship between nutritional status, microbiome effects, susceptibility and severity of infections requires further clinical research data to support this claim. While the risk of contracting the virus is not dependent on nutritional status, the degree of severity and response to the pathogen depends on nutritional health. This review thus provides a rationale that the gut microbiota partially mediates the consequences of SARS-CoV-2 on the host’s immune response and may therefore be a COVID-19 treatment and prevention target.

## Figures and Tables

**Table 1:** The source of various nutrients and diets and their effects on health.

Type of Diet/Nutrient	Source	Characteristics
**Mediterranean diet**	Vegetables, fruit, nuts, legumes, unprocessed cereals	Reduces insulin resistance.
**Western diet**	Red meat, processed foods, sugary drinks	Increases frequency of metabolic diseases and systemic low-grade inflammation. Impairs adaptive immune system.
**Fermented foods**	Cereals, milk, fruits, vegetables, meat	Antioxidant activity.
	Kimchi	Anti-obesity, anti-cancer, anti-diabetic and antioxidant.
	Sauerkraut	Anti-obesity, anti-cancer and antioxidant.
**Fast foods**	Take away restaurants	Reduces *lactobacilli*.
**Vitamin A**	Carrot, sweet potato, green leafy vegetables	Sustains T cells.
**Vitamin C**	Orange, strawberry, mango, red peppers, broccoli, lemon, vegetables	Anti-inflammatory and antioxidant.
**Vitamin D**	Sunlight	antibody-secreting cell functions, Increases anti-inflammatory cytokines and decreases pro-inflammatory cytokines.
**Vitamin E**	Vegetable oils, nuts, seeds, broccoli, spinach	Anti-inflammatory and antioxidant
**Zinc**	Beans, lentils, nuts, sesame, pumpkin seeds, red meat, poultry	Regulates pro-inflammatory responses via NF-κβ
